# Evaluating Compliance to Intra-operative Syndesmosis Stress Testing During Ankle Fracture Fixation: Are We Stressing It?

**DOI:** 10.7759/cureus.92617

**Published:** 2025-09-18

**Authors:** Adam Khan Rahim, Hassan Imtiaz, Abdullah Durrani, Thet Paing Oo, Kunjan Barot, Georgios Kouklidis, Arsallan Karim, Anuraag Sangar

**Affiliations:** 1 Trauma and Orthopaedics, University Hospitals Dorset, Poole, GBR; 2 Orthopaedics, University Hospitals Dorset, Poole, GBR

**Keywords:** ankle x-ray, british orthopaedic association standard for trauma (boast), foot and ankle surgery, quality improvement and patient safety, syndesmosis

## Abstract

Introduction

Ankle fractures are common injuries, and a proportion are complicated by syndesmotic instability. If left unrecognised or untreated, such instability can lead to poor functional outcomes, chronic pain and long-term joint degeneration. Intra-operative stress testing plays a vital role in detecting these injuries, and both radiographic evidence and clear documentation are key to ensuring safe and transparent surgical practice.

Methods

We conducted a retrospective closed-loop audit at a district general hospital to evaluate compliance documentation for intra-operative syndesmosis stress testing during ankle fracture fixation. Target compliance was set at 80% for both radiographic evidence saved and documentation in operative notes. The first audit cycle (November 2023-April 2024) identified gaps in practice and was followed by targeted educational interventions, including posters and dedicated teaching sessions. A second audit cycle was then performed (April-July 2025). Statistical comparison between the two cycles was performed using the Chi-squared test.

Results

Eighty-nine patient records were included in the first cycle and 50 in the second. Initial compliance for radiological evidence saved was 55% (n=49), and documentation was 71% (n=63). After intervention, both domains improved to 82% (n=41), achieving the audit target. The increase in radiological evidence saved was statistically significant (p=0.001), while the improvement in documentation was not (p=0.14). Importantly, the proportion of cases with no evidence saved fell from 25% (n=22) to 6% (n=3).

Conclusion

Targeted, low-cost interventions significantly improved compliance with British Orthopaedic Association Standard for Trauma (BOAST) standards for intra-operative syndesmosis testing. Embedding systematic stress testing, radiographic archiving and operative note documentation into routine practice enhances patient safety, reduces medico-legal risk and supports long-term ankle stability.

## Introduction

Ankle fractures are defined as fractures of the malleoli. These fractures have an incidence of around 180 per 100,000 people per year and are the second most common fractures in adults (9%) after femoral neck fractures [1]. The ankle joint is a hinged synovial joint, which allows movement in one plane, resulting in dorsiflexion and plantar flexion of the foot. The joint is formed by the articulation between the distal tibia, distal fibula and talus. It has three malleoli: the lateral malleolus of the distal fibula, and the medial and posterior malleoli of the distal tibia [2]. The ankle syndesmosis is a fibrous joint connecting the distal tibia and fibula and is comprised of three components: the interosseous tibiofibular ligament, the anterior inferior tibiofibular ligament and the posterior inferior tibiofibular ligament [3]. Of all ankle fractures, 10% are associated with a syndesmotic injury, which requires surgical fixation [4] in order to prevent the development of post-traumatic osteoarthritis [5].

Stability of the ankle syndesmosis is assessed intra-operatively following fixation of the fracture(s), and if found to be disrupted, it requires surgical fixation, which can be achieved either through tight rope or screw fixation [6]. Assessment of syndesmotic stability intra-operatively can be achieved by means of either an external rotation stress test (ESRT) or a lateral hook stress test (LHST) [7].

ESRT involves taking a mortise view of the ankle in plantigrade position with concomitant external rotation forces applied, and this is compared to a mortise view in neutral position with any forces applied. An increase in tibiofibular clear space or medial tibiotalar clear space is taken to be indicative of syndesmotic injury. The LHST is performed by taking a mortise view of the ankle in plantigrade position and applying a lateral distraction force on the fibula by grasping it with a bone hook and pulling laterally. An increase in tibiofibular clear space or medial clear space is considered to be reflective of syndesmotic injury [8].

British Orthopaedic Association Standard for Trauma (BOAST) guidelines recommend that surgical fixation should be aimed at achieving reduction and stabilisation of the ankle mortise, followed by assessment of the syndesmosis and stabilisation if unstable. Intra-operative radiographs should be obtained to confirm reduction [9].

We conducted a closed-loop clinical audit at our trauma unit at a district general hospital to assess the compliance of intra-operative syndesmosis stress following ankle fracture fixation, followed by implementation of targeted interventions and evaluation of their impact in observed clinical practice.

## Materials and methods

This was a retrospective, closed-loop audit conducted at our trauma unit in a district general hospital setting in the United Kingdom. Patients presenting with ankle fractures falling under the Weber B or C classification, or those with a Maisonnaive injury undergoing surgical fixation were included. Patients under the age of 18 years, those with open fractures or those who did not fall into the aforementioned fracture classifications were excluded. Patients were identified from Electronic Clinical and Management Information Software (eCaMIS), a software solution that manages patient administrative details.

The audit standards were based on BOAST guidelines for ankle fractures [9] and evaluated whether radiological evidence of intra-operative syndesmotic stability assessment was saved, along with documentation of this on the operation notes. Following discussion with the Foot and Ankle Consultants, an acceptable target compliance of 80% was agreed upon. These standards are presented in Table 1.

**Table 1:** Audit standards

Standard	Target compliance (%)
Radiological evidence of intra-operative syndesmosis stability saved	80
Documented evidence of intra-operative syndesmosis stability on operation notes	80

Data was collected on patient demographics, ankle fracture classification, syndesmotic stability assessment on intra-operative imaging and its documentation on operation notes. The sources utilised for these measured outcomes were Electronic Patient Records (EPR) and Picture Archiving and Communication System (PACS). Documentation of stress testing was checked on operative notes available on EPR, while intra-operative radiographs of syndesmotic stability assessment, either via LHST or ERST, were assessed on the PACS system and evaluated independently by a senior foot and ankle associate specialist to confirm whether it met the standard criteria.

The first audit cycle was undertaken between November 1, 2023, and April 1, 2024. Gaps in compliance against audit standards were identified and presented at the local clinical governance meeting, followed by agreement to aim for improved compliance. To achieve this, a visual reminder in the form of a poster (Appendices) was put up in relevant clinical areas, including the orthopaedic team on-call room and theatre board rooms. Furthermore, a dedicated PowerPoint presentation (Microsoft Corporation, Redmond, WA) (Appendices) by an associate specialist foot and ankle surgeon was delivered to the orthopaedic team, focusing on the importance of syndesmosis stress testing intra-operatively and its evidence, both in the form of radiological and operative note documentation. Following the aforementioned educational interventions, the subsequent audit cycle was conducted between April 1 and July 1, 2025.

Data was collected, stored and analysed using Microsoft Excel (Microsoft Corporation). Patient identifiers were removed from the datasheet prior to final analysis. Chi-squared test was employed to statistically compare the results of the first and second loop results, with a p-value of less than 0.05 being set as the mark of significance.

## Results

Eighty-nine patient records met the inclusion criteria and were evaluated in the first audit cycle. Of these, Weber B fractures were noted to be the most common fracture type, accounting for 83% (n=74). Furthermore, 55% (n=49) had radiological evidence of intra-operative syndesmosis stress testing saved, while 71% (n=63) had documentation of this assessment on operation notes. For 25% (n=22) of cases, neither radiological nor documented evidence was saved. The results of the second audit cycle are given in Table 2.

**Table 2:** First audit cycle result (N=89)

Audit standard(s)	Observed compliance (%)	Targeted compliance (%)
Radiological evidence of intra-operative syndesmosis stability saved	55 (n=49)	80
Documented evidence of intra-operative syndesmosis stability on operation notes	71 (n=63)	80

Following the implementation of targeted educational interventions, a second audit cycle evaluating 50 cases was conducted. Compliance for both saving radiological evidence of intra-operative syndesmosis stress testing and its documentation on operative notes increased to 82% (n=41). Weber B fractures still remained the most common fracture type at 56% (n=28). For 6% (n=3) of cases, no radiological or documented evidence was saved. The results of the second audit cycle are given in Table 3.

**Table 3:** Second audit cycle results (N=50)

Audit standard(s)	Observed compliance (%)	Targeted compliance (%)
Radiological evidence of intra-operative syndesmosis stability saved	82 (n=41)	80
Documented evidence of intra-operative syndesmosis stability on operation notes	82 (n=41)	80

The second audit cycle showed improvements in both domains: an increase in compliance for radiological evidence of stress testing from 55% to 82%, with documentation on operative notes improving from 71% to 82%. The educational interventions managed to achieve target compliance for both audit standards.

Statistical analysis showed that the improvement in saving radiological evidence between the two audit cycles was statistically significant (p=0.001), while the change in documented evidence reached a p-value of 0.14. The results are presented in Table 4.

**Table 4:** Statistical analysis *Chi-squared test (p<0.05 considered statistically significant)

	First audit cycle (n=89)		Second audit cycle (n=50)		Chi-squared value	p-value*
	Yes	No	Yes	No		
Radiological evidence saved	49	40	41	9	10.18	0.001
Documented evidence saved	63	26	41	9	2.14	0.14

Following the implementation of targeted interventions, including an educational presentation and poster prompts in trauma and theatre areas, the second audit cycle showed marked improvements. Both radiographic and documented compliance increased to 82%, while the proportion of cases lacking any form of evidence dropped dramatically from 25% to 6%. This reflects not only increased awareness but also likely habit formation among surgical teams.

## Discussion

This audit highlights the importance of intra-operative syndesmosis stability assessment during ankle fracture fixation. Syndesmotic injuries, although present in only around 10% of ankle fractures [4], carry a high risk of long-term morbidity if left undiagnosed or untreated. Failure to assess and stabilise an unstable syndesmosis intra-operatively can result in chronic ankle instability, malreduction, persistent pain and accelerated post-traumatic osteoarthritis, all of which can severely impair patient function and quality of life [6,7]. Clinical studies support the fact that accurate reduction of the syndesmosis plays a crucial role in achieving good clinical outcomes in ankle fracture fixation [10,11]. Inadequate assessment may also increase the likelihood of revision surgery, with associated morbidity and cost implications.

Intra-operative stress tests such as the external rotation stress test (ERST) and lateral hook stress test (LHST) remain the gold standard for evaluating syndesmosis stability [7,12]. The findings of this audit reinforce the necessity of not only performing these tests but also saving corresponding radiographic images. Saving intra-operative stress test imaging serves multiple purposes: it provides objective confirmation of adequate assessment, enhances transparency and protects both patient and surgeon in the medico-legal setting. Documentation alone, without radiological evidence, can be challenged; therefore, archiving stress images improves accountability and strengthens trust in surgical practice. The significant improvement in compliance across audit cycles demonstrates that even low-cost educational interventions can substantially improve adherence to best practice standards.

The medico-legal implications of inadequate documentation cannot be underestimated. Litigation arising from missed or inadequately managed ankle syndesmotic injuries is costly, with malpractice claims in orthopaedic foot and ankle surgery often ranking among the highest in orthopaedics [13]. In the United Kingdom, orthopaedic claims represent a major proportion of NHS Resolution payouts, costing around 90 million pounds per year [14]. These figures highlight the importance of ensuring adherence to recommended intra-operative assessment of ankle syndesmosis stability [9], and transparent record-keeping is not only clinically essential but also economically prudent.

This article demonstrates how the implementation of simple educational interventions can achieve significant improvements in both imaging and documentation of intra-operative stress testing. Importantly, the reduction in cases with no evidence saved, from 25% to 6%, represents a significant improvement in safeguarding patients from missed injuries and in protecting clinicians from medico-legal vulnerability.

Overall, this audit reinforces that systematic intra-operative syndesmosis testing and thorough documentation should be embedded as routine practice. Regular audit cycles, coupled with targeted education, ensure that standards are upheld, transparency is maximised and both patient safety and medico-legal protection are enhanced.

At the end, we would like to address the limitations of this study. The second audit cycle included only 50 cases, which may reduce the statistical power of comparisons between the initial and post-intervention groups. As the audit was conducted within a single hospital's orthopaedic department, findings may reflect local practices and may not be representative of national or international standards. While the audit evaluated the presence or absence of stress testing documentation and imaging, it did not assess the accuracy, quality or technique of the hook or external rotation stress tests themselves, which could vary between surgeons. The audit focused on process compliance rather than patient-centred outcomes. It does not assess whether improved documentation or imaging led to better syndesmotic reduction, fewer reoperations or improved functional outcomes. While the educational intervention yielded short-term improvements in compliance, the long-term sustainability of these changes remains unclear, and therefore, further audit cycles down the line would help highlight this domain and are recommended.

## Conclusions

This audit demonstrated that simple yet cost-effective educational interventions can significantly improve the compliance of intra-operative stress testing and its documentation of ankle syndesmosis during ankle fracture fixation. This reflects greater awareness among surgeons and promotes safer clinical practice. Systematic stress testing and transparent documentation are not only essential for accurate detection and management of syndesmotic injuries but also provide critical protection in the medico-legal context.

Sustaining these improvements requires regular audit cycles, ongoing education and multidisciplinary engagement. By embedding rigorous intra-operative assessment and record-keeping into everyday practice, trauma teams can enhance patient safety, reduce complications and foster a more accountable and transparent surgical environment.

## Figures and Tables

**Figure 1:**
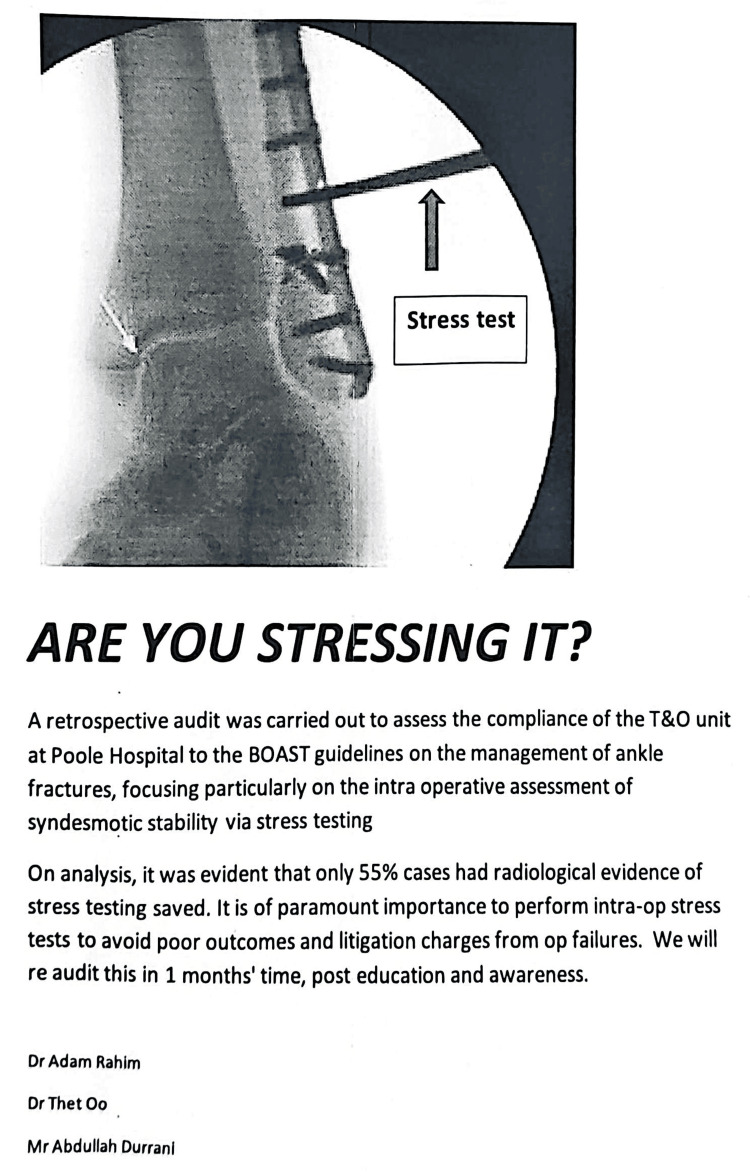
Quality improvement poster: enhancing compliance in syndesmosis stress test documentation and imaging

**Figure 2:**
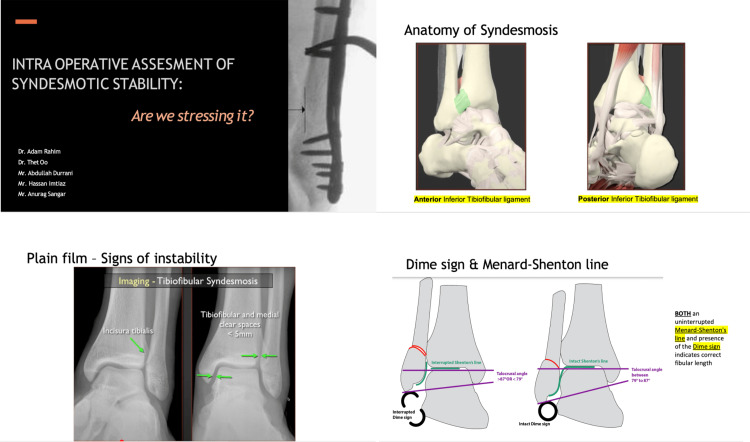
Implementing change through audit: a presentation on improving syndesmosis stress test practice Slides 1-4

**Figure 3:**
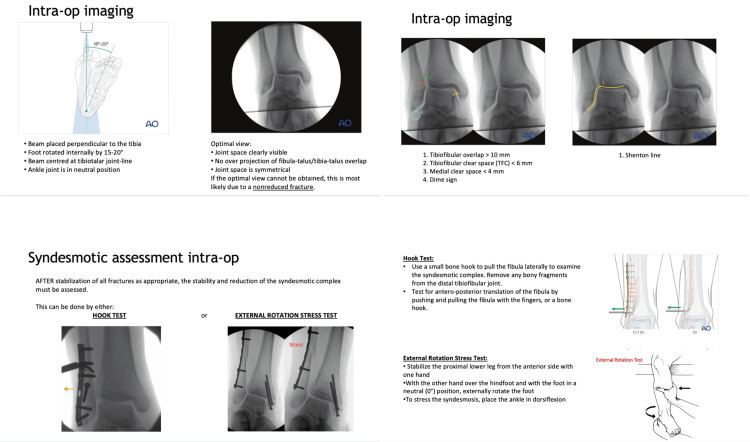
Implementing change through audit: a presentation on improving syndesmosis stress test practice Slides 5-8

**Figure 4:**
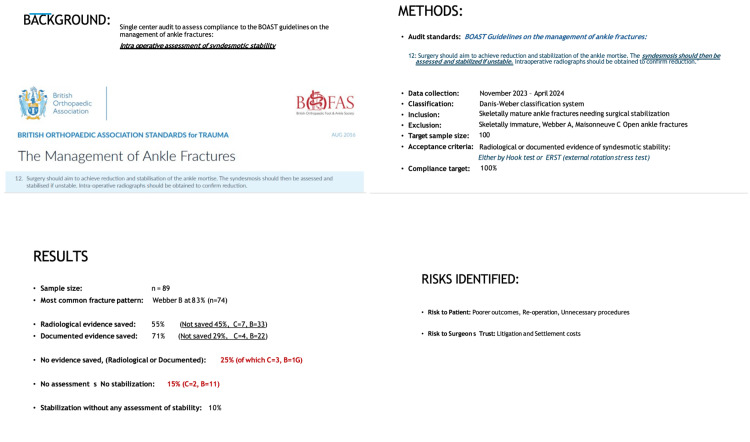
Implementing change through audit: a presentation on improving syndesmosis stress test practice Slides 9-12

**Figure 5:**
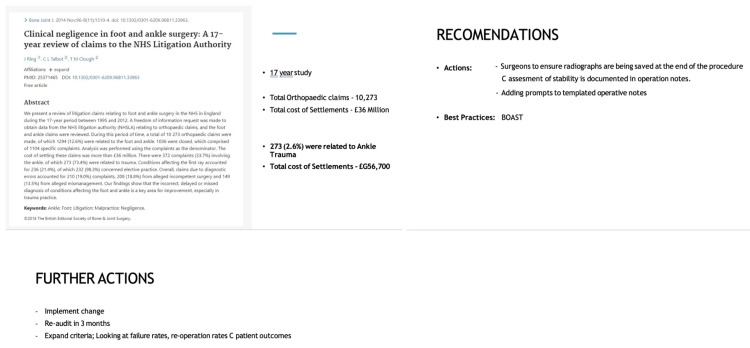
Implementing change through audit: a presentation on improving syndesmosis stress test practice Slides 13-15

## Appendices

Figure 1 presents the poster displayed in clinical areas to increase awareness.

Figures 2-5 present the educational presentation to orthopaedic team members to increase awareness about intra-operative syndesmotic testing and optimal techniques.
